# Obesity Enhances Antioxidant Capacity and Reduces Cytokine Levels of the Spleen in Mice to Resist Splenic Injury Challenged by *Escherichia coli*

**DOI:** 10.1155/2020/5948256

**Published:** 2020-02-11

**Authors:** Xuchu Gu, Zhiyu Ma, Jing Fang, Dongjie Cai, Zhicai Zuo, Shuang Liang, Hengmin Cui, Junliang Deng, Xiaoping Ma, Zhihua Ren, Yi Geng, Ming Zhang, Gang Ye, Yue Xie, Liping Gou, Yanchun Hu

**Affiliations:** Key Laboratory of Animal Disease and Human Health of Sichuan Province, College of Veterinary Medicine, Sichuan Agricultural University, Chengdu Sichuan Province 611130, China

## Abstract

Obese mice exhibited more lymphocytes in the bronchoalveolar lavage fluid and milder lung injury after *Escherichia coli* (*E. coli*) infection. However, it remained unclear whether the spleen contributed to the effect of obese mice with infection. The study was purposed to reveal the histopathological changes of the spleen caused by oxidative stress and inflammation in diet-induced obesity (DIO) mice challenged by *Escherichia coli*. After infection, the spleen tissues were obtained in normal and DIO mice at 0 h (uninfected), 12 h, 24 h, and 72 h postinfection. Results revealed that DIO mice have higher contents of resistin, TNF-*α*, IL-6, and IL-1*β* in the spleen than normal mice and lower concentrations of GSH-Px, SOD, and CAT and higher MDA than normal mice. After an intranasal drip of *E. coli*, the activities of GSH-Px, SOD, and CAT in the DIO mice were elevated and the content of MDA declined. The activities of SOD and CAT in the normal mice declined, and the content of MDA was elevated. Moreover, the contents of TNF-*α*, IL-6, and IL-1*β* in the spleen declined in DIO mice at 24 and 72 h, although the contents of leptin, resistin, TNF-*α*, IL-6, and IL-1*β* were elevated at 12 h. The contents of resistin, TNF-*α*, IL-6, and IL-1*β* were elevated in normal mice at 12 and 24 h. Those results indicated that obesity elevated splenic oxidation and inflammatory levels, but it enhanced antioxidant capacity and reduced cytokine levels of the spleen in mice to resist splenic injury after an intranasal drip of *E. coli*.

## 1. Introduction

Obesity is a complex chronic disorder with multifactorial etiology, involving genetics, hormones, diet, and environments [1]. Emerging data indicated that obesity is closely related to infectious diseases and obesity increased susceptibility to bacterial infections [2]. Obesity was recognized as a significant risk factor for pneumonia with increased incidence and severity of disease [3]. Fascinatingly, Wan et al. [4] have reported that the obese mice exhibited more lymphocytes in the bronchoalveolar lavage fluid and milder lung injury than normal mice under the case of nonfatal pneumonia caused by *E. coli*, suggesting that obesity can improve immune responses against bacterial pneumonia. The DIO mice also presented a delayed pulmonary inflammatory response and oxidative stress in pneumonia induced by *E. coli* infection [5]. In addition, the previous study found that the changes of hepatic histopathological damage, oxidative damage, and higher levels of cytokines (TNF-*α*, IL-6, and resistin) showed to be less severe in the DIO mice than in the normal mice following *E. coli* pneumonia [6]. However, the effect on the spleen is unclear at present.

The spleen, the largest immune organ in the animals, is mainly involved in filtering the blood, recycling iron, capturing pathogens, and activating adaptive immune response [7]. Altunkaynak et al. [8] showed spleen enlargement in obese SD rats, and sinusoidal dilatation and intracellular or intercellular deposits were found in the spleen. Also, the study exhibited an increased number of large mononuclear cells in the white pulp, moderate necrosis, and congestion of the red pulp in mice after bacterial infection [9]. The change of spleen structure affects the immune function of the organism.

Oxidative damage and local inflammatory reaction are important pathological mechanisms of tissue damage. Oxidative stress is defined by an imbalance between increased levels of reactive oxygen species (ROS) and a low activity of antioxidant mechanisms, and it plays an important role in physiological and pathological processes of the body. In obesity, adipose tissue exhibited the lower activities of antioxidant enzymes (CAT, SOD, and GSH-Px) and the higher levels of cytokines (TNF-*α*, IL-1*β*, and IL-6) and ROS generation [10]. Moreover, the *Escherichia coli* LPS-treated rats developed oxidative stress [11]. People with acute pneumonia exhibited an increased oxidative stress [12]. Also, the study showed the increased secretion of TNF-*α* and IL-6 in the spleen after bacterial infection [13].

Importantly, very little information regarding the oxidative stress and inflammation of the spleen in obese and normal subjects after *E. coli* infection is available so far. To explore this knowledge gap, the present study was carried out to discuss the changes of the inflammatory responses, oxidative stress status, and pathology in the spleen of the normal and DIO mice with nonfatal pneumonia induced by *E. coli*. Those results would provide a new view to understand the role of the spleen in obesity against pneumonia.

## 2. Materials and Methods

All care and study protocols on animals were performed according to the Guidelines for the Care and Use of Laboratory Animals and were approved by the Ethics Committee of Sichuan Agricultural University (Approval No. 2012-024, Chengdu, China). The animal experiments took place in the clinical laboratory at Sichuan Agricultural University.

### 2.1. Animals

Male Kunming mice (24 ± 1 days, 12-15 g) were obtained from the Dashuo Animal Center (Chengdu, China) and housed under Specific Pathogen-Free (SPF) raising conditions. After one week of adaptation to the environment, all mice were primarily divided into two groups: one is the normal (N) group (*n* = 128) and the other is the diet-induced obesity (DIO) group (*n* = 128). The body weight of the mice in each feeding group was measured weekly, and DIO mice could be obtained after feeding the commercial feed diets for 8 weeks from the Dashuo Animal Center (Chengdu, China) as described previously [4, 14]. During the experiment, all mice were kept in constant temperature environment at 24°C. Food and water were supplied *ad libitum*.

### 2.2. Groups and *E. coli* Infection

After continuous feeding for 8 weeks on the abovementioned diets, the mice were divided into 4 groups: N-PBS (*n* = 64), N-*E. coli* (*n* = 64), DIO-PBS (*n* = 64), and DIO-*E. coli* (*n* = 64). By feeding formula, the obese index of mice used in high-fat groups is higher than 20%. Additionally, serum triglyceride and total cholesterol contents (A110-1 and A111-1, Nanjing Jiancheng Bioengineering Institute, China) were examined. The mice in N-*E. coli* and DIO-*E. coli* were anesthetized with ether and challenged intranasally with 40 *μ*L of bacterial suspension containing approximately 10^9^ CFUs of *E. coli* which was provided by the Veterinary Medical Laboratory of Sichuan Agricultural University (Chengdu, China) as described previously [4, 5]. And the same dose of PBS was given to the mice in N-PBS and DIO-PBS by the same method.

(1) $$\begin{array}{c} \text{Obese index}=\frac{\text{individual weight of DIO}-\text{average weight of normal}}{\text{average weight of normal}}\times 100\%. \end{array}$$

### 2.3. Body Weight and Lee's Index

Body weight was measured after 8 weeks of high-fat diet, and body length was also measured to calculate Lee's index.

(2) $$\begin{array}{c} \text{Le}{\text{e}}^{\text{'}}\text{s index}=\frac{\sqrt[{3}]{\text{body weight}\left(\text{g}\right)}}{\text{length}\left(\text{cm}\right)}\times 1000. \end{array}$$

### 2.4. Spleen Index

Body weight of the normal and DIO mice was determined at 0 h (uninfected), 12 h, 24 h, and 72 h postinfection. After mice were sacrificed by cervical dislocation, spleens were removed at the different time points after infection and weighed, and the spleen index was calculated by the following formula:

(3) $$\begin{array}{c} \text{Spleen index}=\frac{\text{spleen weight}\left(\text{mg}\right)}{\text{body weight}\left(\text{g}\right)}. \end{array}$$

### 2.5. Preparation of Spleen Homogenates

At 0 h (uninfected), 12 h, 24 h, and 72 h postinfection, eight mice in each group were sacrificed and the spleen tissues were collected for the detection of cytokine levels by the enzyme-linked immunosorbent assay (ELISA) and antioxidant defense system assays by a biochemical method. The spleen was added to PBS according to the proportion of 1 : 9; then, a tissue homogenate was made in the ice bath using a tissue homogenizer and centrifuged at 3,000 rpm for 10 min at 4°C to obtain a supernatant.

### 2.6. Antioxidant Defense System Assays

The spleen supernatant was used for the detection of the content of MDA and the activities of GSH-Px, CAT, and SOD, respectively, using kits according to the manufacturer's instructions (A003-1, A005, A007-1, and A001-3, Nanjing Jiancheng Bioengineering Institute, Nanjing, China). The absorbance of the spleen supernatant was measured by a spectrophotometric assay at 532 nm and 95°C for MDA, 412 nm and 37°C for GSH-Px, 450 nm for SOD, and 405 nm for CAT. The values were expressed as nmol/mg protein for MDA and units (U)/mg protein for GSH-Px, CAT, and SOD.

### 2.7. Measurement of Spleen Cytokines

The concentrations of leptin, resistin, TNF-*α*, IL-6, and IL-1*β* in the spleen homogenates were measured by mouse ELISA kits (H174, H175, H052, H007, and H002, Nanjing Jiancheng Bioengineering Institute), according to the manufacturer's instructions. The cytokine concentrations in the spleen were calculated from standard curves by ELISA Calc software.

### 2.8. Hematoxylin-Eosin and Masson Trichrome Staining

At 0 h, 12 h, 24 h, and 72 h of the experiment, eight mice in each group were sacrificed and the spleen tissues were observed and photographed. And then, the spleen tissues were fixed immediately in 4% paraformaldehyde and routinely processed in paraffin. Dehydrated, transparent, paraffin-embedded, paraffin sections of 4 to 6 *μ*m were cut and stained with hematoxylin and eosin (HE) and Masson for histological observations.

### 2.9. Statistical Analysis

Data are expressed as the means ± standard deviations (SD). Statistical analyses were performed to compare between obese and normal groups using one-way analysis (LSD or Dunnett T3) of variance by SPSS22.0 (IBM Corp., Armonk, NY, USA). *p* values < 0.05 were considered to be significant. Moreover, to normalize the baseline of different status, all parameters were calculated as ratios (infected/uninfected), which could not only reflect the response intensity of DIO and normal mice to infection at different times but also show the change trend of spleen targets in the process of infection.

## 3. Results

### 3.1. Changes of Clinical Symptoms following *E. coli* Challenge

At 0 h (uninfected), the mice in each group had a good mental state, sensitive reaction, and normal drinking and appetite. After infection, there were no significant changes in the mental status, feeding status, and activity status of the mice in N-PBS and DIO-PBS groups. However, the mice in N-*E. coli* and DIO-*E. coli* groups had symptoms of poor mental status, decreased or stopped feeding, and immobility, especially at 12 h. No death occurred in all groups.

### 3.2. Higher Body Weight, Lee's Index, and Serum TG and TC Levels in DIO after 8 Weeks

**Figure 1:**
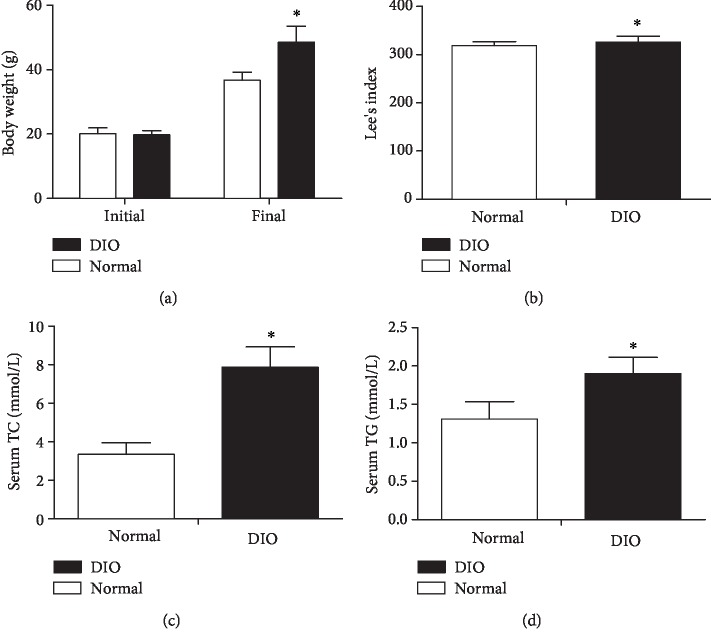
The changes of body weight (a), Lee's index (b), serum TC (c), and serum TG (d) in the DIO mice after 8 weeks of high-fat diet. Values are expressed as means ± SD (*n* = 8 mice per group). Asterisks indicate significant differences between DIO and normal groups after 8 weeks (*p* < 0.05).

In Figure 1, after 8 weeks of high-fat diet, the obese index of mice in high-fat groups was more than 20% and significantly higher than that of the normal mice (*p* < 0.05). Furthermore, the DIO mice exhibited a higher Lee's index and serum TG and TC levels than the normal mice (*p* < 0.05).

### 3.3. Difference in Spleen Weight and Spleen Index following *E. coli* Challenge

**Figure 2:**
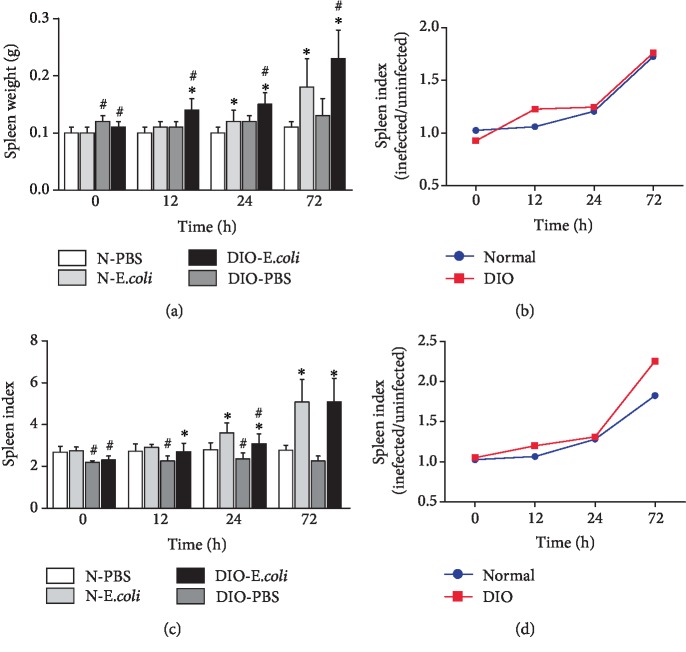
Changes of spleen weight and spleen index after infection with *E. coli*: (a) spleen weight, (b) spleen weight (infected/uninfected), (c) spleen index, and (d) spleen index (infected/uninfected). Values are expressed as means ± SD (*n* = 8 mice per group). Asterisks indicate significant difference between N-*E. coli* and N-PBS, as well as DIO-*E. coli* and DIO-PBS (*p* < 0.05). Octothorps indicate significant difference between DIO-PBS and N-PBS, as well as DIO-*E. coli* and N-*E. coli* (*p* < 0.05).

Compared with the normal group, the spleen weight in the DIO group was higher at 0 h, 12 h, 24 h, and 72 h (*p* < 0.05). At 12-72 h after infection with *E. coli*, the spleen weight in N-*E. coli* was significantly higher than that in N-PBS (*p* < 0.05); the spleen weight in DIO-*E. coli* was also significantly higher than that in DIO-PBS (*p* < 0.05). And the change range of spleen weight in DIO mice was larger than that in normal mice at 12 h, but it tends to be consistent at 24 and 72 h (Figures 2(a) and 2(b)).

The spleen index in DIO mice was lower than that in normal mice across all time points (*p* < 0.05). At 12-72 h after infection with *E. coli*, the spleen index in N-*E. coli* was significantly higher than that in N-PBS (*p* < 0.05); the spleen index in DIO-*E. coli* was also significantly higher (*p* < 0.05) than that in DIO-PBS. Interestingly, the increased extent of the spleen index of DIO mice was more than that of normal mice (Figures 2(c) and 2(d)).

### 3.4. The Changes on the State of Oxidative Stress following *E. coli* Infection

**Figure 3:**
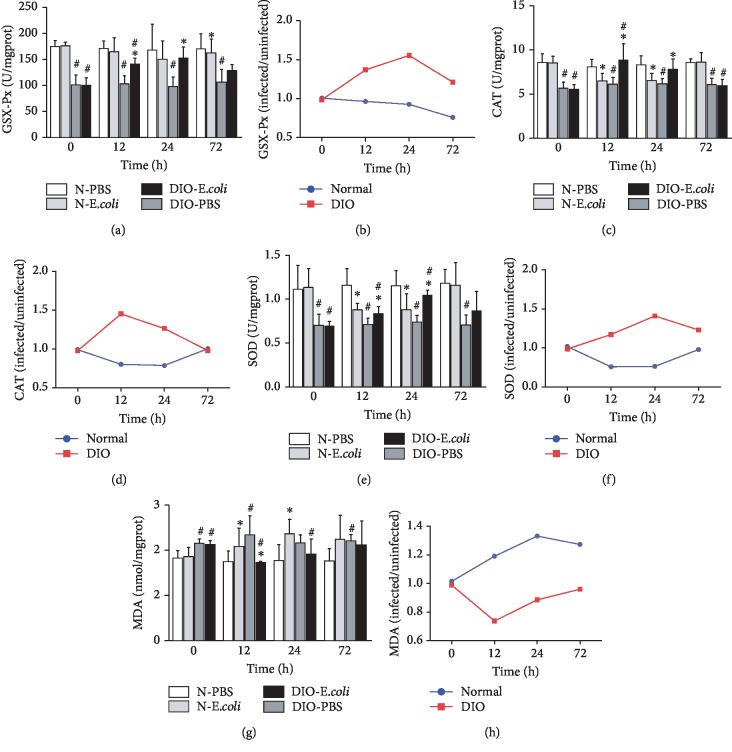
The changes on the state of oxidative stress following *E. coli* infection: (a) GSH-Px activities, (b) GSH-Px (infected/uninfected), (c) SOD activities, (d) SOD (infected/uninfected), (e) CAT activities, (f) CAT (infected/uninfected), (g) MDA concentrations, and (h) MDA (infected/uninfected). Values are expressed as means ± SD (*n* = 8 mice per group). Asterisks indicate significant difference between N-*E. coli* and N-PBS, as well as DIO-*E. coli* and DIO-PBS (*p* < 0.05). Octothorps indicate significant difference between DIO-PBS and N-PBS, as well as DIO-*E. coli* and N-*E. coli* (*p* < 0.05).

To evaluate the state of oxidative stress in the spleen, the content of MDA and the activities of GSH-Px, SOD, and CAT were measured. The activity of GSH-Px in DIO-PBS was lower than that in N-PBS from 0 h to 72 h (*p* < 0.05). The activity of GSH-Px in N-*E. coli* was significantly lower than that in N-PBS (*p* < 0.05) at 72 h. However, the activity of GSH-Px in DIO-*E. coli* was higher (*p* < 0.05) than that in DIO-PBS at 12 h and 24 h (Figure 3(a)). The activity of GSH-Px in DIO mice showed an uptrend from 0 to 24 h but downtrend from 24 to 72 h. The activity of GSH-Px in normal mice has been decreasing during the infection (Figure 3(b)).

Compared with N-PBS, the activity of SOD in DIO-PBS was lower across all time points (*p* < 0.05). At 12 h and 24 h after infection with *E. coli*, the activity of SOD in N-*E. coli* was significantly lower than that in N-PBS (*p* < 0.05), while the activity of SOD in DIO-*E. coli* was significantly higher (*p* < 0.05) than that in DIO-PBS (Figure 3(c)). The activity of SOD in DIO mice showed an uptrend from 0 to 12 h but downtrend from 12 to 72 h. The activity of SOD in normal mice showed a downtrend from 0 to 12 h and uptrend from 12 to 72 h (Figure 3(d)).

The activity of CAT in DIO-PBS was lower than that in N-PBS at all time points (*p* < 0.05). The activity of CAT in N-*E. coli* was significantly lower than that in N-PBS at 12 h and 24 h (*p* < 0.05), while CAT in DIO-*E. coli* was significantly higher (*p* < 0.05) than that in DIO-PBS (Figure 3(e)). The activity of CAT in DIO mice showed an uptrend from 0 to 24 h but downtrend from 24 to 72 h. The activity of CAT in normal mice showed a downtrend from 0 to 12 h and uptrend from 12 to 72 h (Figure 3(f)).

After infection, the antioxidant capacity in normal mice was decreased. Conversely, it increased in DIO mice. The content of MDA in DIO mice was higher than that in normal mice from 0 h to 72 h (*p* < 0.05). The MDA content in N-*E. coli* was significantly higher than that in N-PBS at 12 h and 24 h (*p* < 0.05); MDA content in DIO-*E. coli* was lower (*p* < 0.05) than that in DIO-PBS at 12 h (Figure 3(g)). The content of MDA in DIO mice showed an uptrend from 0 to 24 h but downtrend from 24 to 72 h. The content of MDA in normal mice showed a downtrend from 0 to 12 h and uptrend from 12 to 72 h (Figure 3(h)).

### 3.5. The Changes on Cytokines following *E. coli* Infection

**Figure 4:**
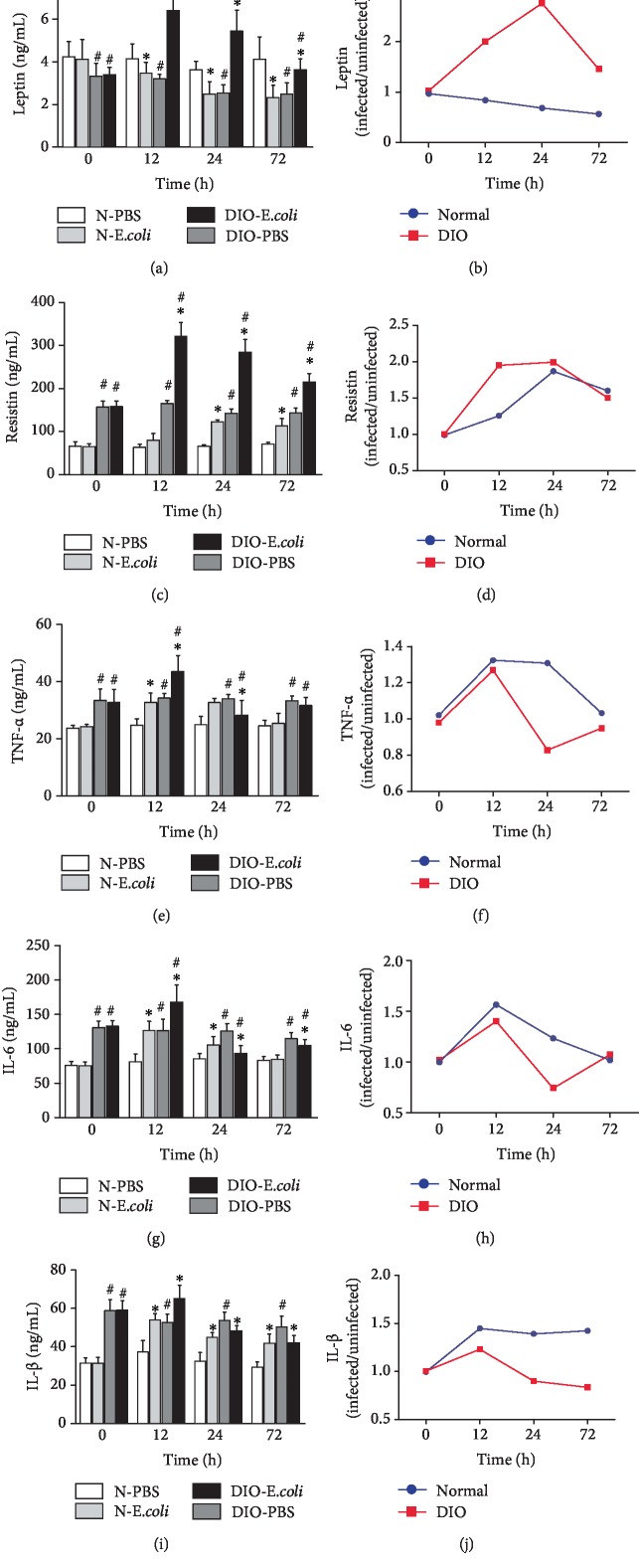
The changes on cytokines following *E. coli* infection: (a) leptin, (b) leptin (infected/uninfected), (c) resistin, (d) resistin (infected/uninfected), (e) TNF-*α*, (f) TNF-*α* (infected/uninfected), (g) IL-6, (h) IL-6 (infected/uninfected), (i) IL-1*β*, and (j) IL-1*β* (infected/uninfected). Values are expressed as means ± SD (*n* = 8 mice per group). Asterisks indicate significant difference between N-*E. coli* and N-PBS, as well as DIO-*E. coli* and DIO-PBS (*p* < 0.05). Octothorps indicate significant difference between DIO-PBS and N-PBS, as well as DIO-*E. coli* and N-*E. coli* (*p* < 0.05).

The concentration of leptin in the spleen of DIO-PBS was significantly lower than that in N-PBS across all time points (*p* < 0.05). At 12 h, 24 h, and 72 h, the concentration of leptin in N-*E. coli* was significantly lower than that in N-PBS (*p* < 0.05); however, the concentration of leptin in DIO-*E. coli* was higher (*p* < 0.05) than that in DIO-PBS at 12 h, 24 h, and 72 h (Figure 4(a)). The concentration of leptin in DIO mice increased from 0 to 24 h but decreased from 24 to 72 h. The concentration of leptin in normal mice decreased from 0 to 72 h (Figure 4(b)).

The concentration of resistin in DIO-PBS was higher than that in N-PBS across all time points (*p* < 0.05). Resistin concentration in N-*E. coli* or DIO-*E. coli* was higher than that in N-PBS or DIO-PBS (*p* < 0.05) from 12 h to 72 h, respectively (Figure 4(c)). It is worth noting that the increased extent of resistin in DIO mice was more than that in normal mice (Figure 4(d)).

The concentration of TNF-*α* in DIO-PBS was higher than that in N-PBS at all time points (*p* < 0.05). The concentration of TNF-*α* in N-*E. coli* or DIO-*E. coli* was significantly higher than that in N-PBS or DIO-PBS, respectively, at 12 h (*p* < 0.05); however, the concentration of TNF-*α* in DIO-*E. coli* was lower than that in DIO-PBS (*p* < 0.05) at 24 h (Figure 4(e)). It is worth noting that the decreased extent of TNF-*α* of DIO mice was more than that of normal mice (Figure 4(f)).

The concentration of IL-6 in DIO-PBS was higher than that in N-PBS at all time points (*p* < 0.05). The concentration of IL-6 in N-*E. coli* was significantly lower than that in N-PBS at 12 h and 24 h (*p* < 0.05); the concentration of IL-6 in DIO-*E. coli* was significantly higher (*p* < 0.05) than that in DIO-PBS at 12 h, and the concentration of IL-6 in DIO-*E. coli* was lower than that in DIO-PBS at 24 h and 72 h (Figure 4(g)). Interestingly, the decreased extent of IL-6 in DIO mice was more than that in normal mice (Figure 4(h)).

The concentration of IL-1*β* in DIO-PBS was higher than that in N-PBS at all time points (*p* < 0.05). At 12 h, 24 h, and 72 h, the concentration of IL-1*β* in N-*E. coli* was higher than that in N-PBS (*p* < 0.05). The concentration of IL-1*β* in DIO-*E. coli* was significantly higher (*p* < 0.05) than that in DIO-PBS at 12 h, and the concentration of IL-1*β* in DIO-*E. coli* was lower than that in DIO-PBS at 24 h and 72 h (Figure 4(i)). It is worth noting that the decreased extent of IL-1*β* of DIO mice was more than that of normal mice (Figure 4(j)).

### 3.6. Differences in Histopathology following *E. coli* Infection

**Figure 5:**
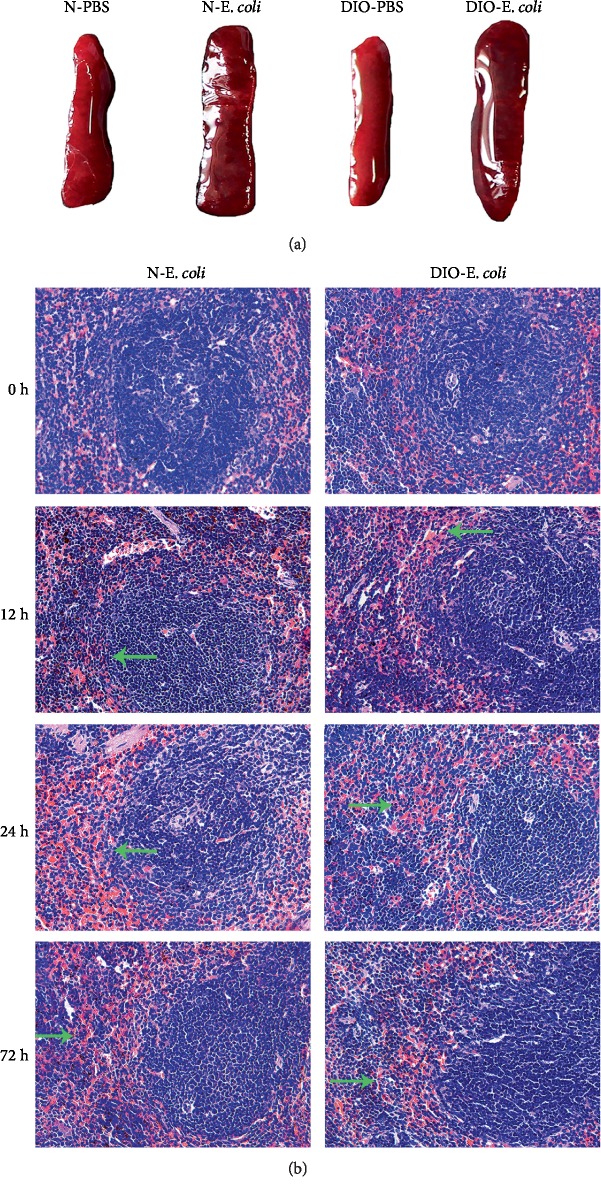
*E. coli*-induced histological changes in normal and DIO mice. (a) Representative spleen anatomy changes following *E. coli* (12 h). (b) H&E staining of spleen sections was visualized via light microscopy to examine spleen architecture. Images were taken at 400x magnification. *n* = 8 mice per group. The arrow points to hyperemia.

As shown in Figure 5(a), the spleen with dark red color and soft texture in N-PBS and DIO-PBS exhibited a normal macroscopic structure. After being challenged with *E. coli*, the spleens of N-*E. coli* and DIO-*E. coli* were dark and volume increased; moreover, the texture became hard.

**Figure 6:**
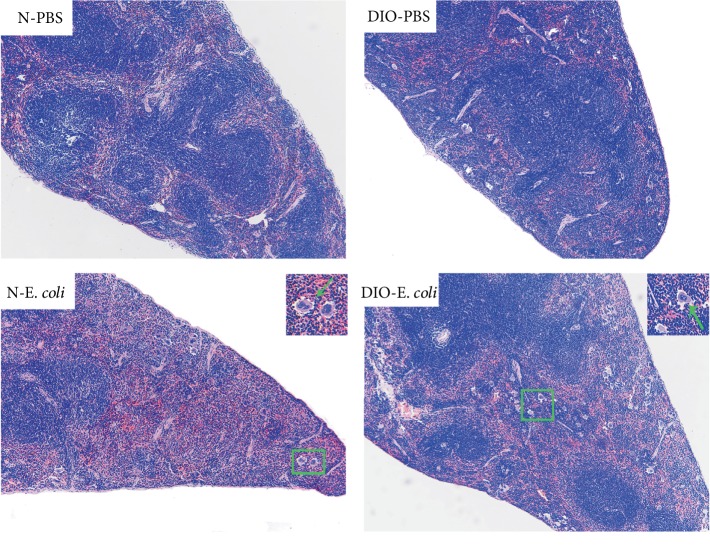
Histological images of spleen tissue at 12 h. Hematoxylin and eosin (H&E) staining of spleen sections was visualized via light microscopy to examine spleen architecture. Images were taken at 100x magnification. *n* = 8 mice per group. The arrow points to multinuclear giant cells.

By microscopic observation, the spleen of N-*E. coli* and DIO-*E. coli* mice had normal histology with clear white and red pulp at 0 h, but slight hyperemia of red pulp was found in DIO-*E. coli*. At 12 h after infection, the red pulp of N-*E. coli* and DIO-*E. coli* mice was seriously hyperemic and the lymphocyte arrangement was looser in white pulp in N-*E. coli*, and an increase in the macrophages and a few multinuclear giant cells (Figure 6) in DIO-*E. coli* was discovered. At 24 h, a small amount of plasma cells were found in the marginal zone of N-*E. coli* mice. There were a large number of macrophages and multinuclear giant cells (MGC) in red pulp and vacuoles in the splenic sinuses, and the lymphocyte arrangement was looser in white pulp. The red pulp of DIO-*E. coli* mice was hyperemic, and a large number of neutrophils were found in the red pulp. At 72 h, there was still slight hyperemia of red pulp in N-*E. coli* and DIO-*E. coli* mice, but the others were not much different from those before infection (Figure 5(b)).

### 3.7. The Change of Spleen Fibrosis after Infection

**Figure 7:**
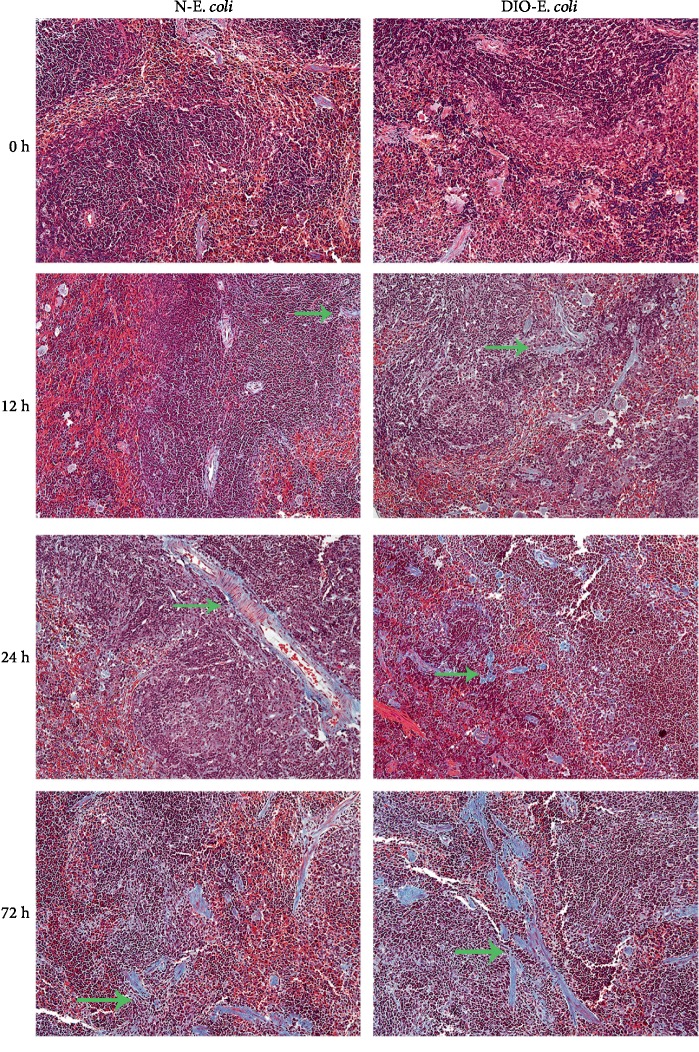
Histological images of spleen tissue (Masson). Masson trichrome staining of spleen sections was visualized via light microscopy to examine the degree of spleen fibrosis. Images were taken at 200x magnification. *n* = 8 mice per group. The arrow points to collagen fibers.

The muscle fiber was red and the collagen fiber was blue. The blue collagen fibers were found in the spleen by the Masson trichrome staining. The spleen was normal in both normal and DIO mice before infection. After being challenged with *E. coli*, the blue collagen fibers were observed in the spleen of N-*E. coli* and DIO-*E. coli* mice under a microscope (as shown in Figure 7). At 24 h and 72 h, a certain amount of blue collagen fibers can be seen under a microscope in the N-*E. coli* and DIO-*E. coli*, especially at 72 h. It is worth noting that DIO-*E. coli* mice had more collagen fiber than N-*E. coli* mice.

## 4. Discussion

Obesity is growing rapidly around the world, and the diseases associated with obesity have been highlighted in recent years, especially pulmonary disease. Recently, studies suggested that obesity plays a beneficial role in *E. coli* pneumonia, manifesting that clinically significant changes in pulmonary inflammatory response may be associated with immune status in obesity [4, 5]. The spleen is the center of cellular immunity and humoral immunity. The previous study indicated that the spleen plays an important role in dampening the inflammatory response and promoting wound repair [15]. It is necessary to determine how the spleen was affected in obesity host with *E. coli* pneumonia.

The mice receiving high-fat diet exhibited higher body weight, Lee's index, and levels of TC and TG than those receiving normal diet, which indicated that the DIO mouse model was established as previously described by Wan et al. [4]. Altunkaynak et al. [8] found that high-fat diet induced splenomegaly in female SD rats fed with high-fat diet for a 3-month duration. However, Trufakin et al. [16] have also found that the spleen weight declined in Wistar rats after giving high-fat diet. Those different outcomes may result from different animal models. In the study, the spleen weight of DIO mice was higher than that of normal mice, which was consistent with other studies [17]. The strength of immune function of mice challenged with *E. coli* can be indicated by the spleen index. Kumagai et al. [18] found that the spleen weight increased after intravenous injection of the LPS. The current study found that *E. coli* helped in increasing the splenic index in both DIO and normal mice and the elevation in DIO mice was stronger than that in normal mice, which indicated that the anti-infection effect of the spleen might depend on increasing the volume, and obesity can enhance the sensitivity of the spleen to *E. coli* infection in mice.

Researches showed that obesity induced oxidative stress and obesity may be a state of chronic oxidative stress, an increase in NO and MDA contents, and a significant decrease in activities of SOD, CAT, and GPx [19, 20]. Gheorghe et al. [21] found that the spleen redox balance in obese mice was broken by reducing GSH levels and the GSH/GSSG ratio and increasing GSSG activity in the spleen, further inducing the changes of spleen immune functions. The present study showed that activities of GSH-Px, SOD, and CAT in DIO mice were lower than those in normal mice and MDA content was higher, consistent with previous studies [22, 23], suggesting that HFD altered the oxidant-antioxidant balance and increased the splenic oxidation level.

It is well known that the oxidative stress is inseparable from the immune function of the body and that it is the key against foreign pathogens [24]. Gor^1^ca et al. [11] found that the splenic TBARS and H_2_O_2_ concentrations increased and GR and GSH levels decreased in rats after *Escherichia coli* LPS administration. Cemek et al. [12] reported that SOD and GPx in the whole blood decreased while MDA and GSH increased in children with acute pneumonia and proved that all antioxidant vitamin activities decreased in children with acute pneumonia. In this study, after *E. coli* infection, the activities of antioxidases, including GSH-Px, SOD, and CAT, all significantly increased in DIO-*E. coli* but decreased in N-*E. coli*. The SOD, CAT, APx, and GSH-Px can keep a low level of ROS and prevent ROS from poisoning cells [25], and these antioxidant enzymes play key roles in the antioxidant mechanisms of the host. Increased levels of activities of SOD, CAT, and GSH-Px were found in DIO-*E. coli* after infection, which might reduce the spleen oxidative damage after infection and enhance the ability to defend against infection. The MDA is the final product of lipid peroxidation, and it is considered an important indicator of oxidative damage to the cellular membrane [26]. In the present study, we found that MDA content in DIO-*E. coli* was lower than that in DIO-PBS, but higher in N-*E. coli* than that in N-PBS after infection, which suggested that the obese mice exhibited stronger regulatory ability than normal mice in the case of infection and obese mice could alleviate oxidative stress and oxidative damage in the spleen through modulating the antioxidant defense system.

In addition to examination of the state of oxidative stress, we also measured the changes of some cytokines in the spleen to assess the inflammatory and immune status, including leptin, resistin, IL-1*β*, IL-6, and TNF-*α*. Obesity is a chronic low-grade inflammation, and the concentrations of resistin [27], leptin [28], and IL-1*β*, as well as TNF-*α* and IL-6 [26, 29], in peripheral blood of obese patients were significantly increased. In our study, increased levels of resistin, TNF-*α*, IL-1*β*, and IL-6 were observed in the spleen in DIO mice, which suggested that the changes of cytokine in the spleen in obesity were indicative of altered immune functions [30].

Leptin, a 167 polypeptide mainly secreted by the adipose tissue, takes part in the regulation of energy metabolism. Moreover, several *in vitro* studies have shown that leptin can upregulate the immune function by proliferation and cytokine production of lymphocytes, phagocytic function, etc. [31, 32]. A recent study showed that leptin could induce secretion of inflammatory cytokines IL-6 and TNF-*α* via JAK2/STAT3 and p38MAPK/ERK1/2 signaling pathways [33]. In the current study, after infection, the level of leptin was significantly increased in DIO-*E. coli* mice and decreased in N-*E. coli* mice.

Resistin, a 12.5 kDa adipocytokine involved in the development of insulin resistance as described elsewhere, takes part in the regulation of energy metabolism [34]. Usually, resistin can increase IL-1*β*, IL-6, and TNF-*α* expression by a multicytokine “resistin pathway” in cells and tissues [35]. IL-6, TNF-*α*, and IL-1*β* are important proinflammatory cytokines in *vivo* and can reflect the severity of inflammation in the body. Interleukin 6 (IL-6) is secreted by T cells and macrophages to stimulate immune response after infection and trauma. IL-6 also plays a role in fighting infection, as IL-6 has been proven in mice to be required for resistance against the bacterium *Streptococcus pneumonia* [36]. TNF-*α*, also produced chiefly by activated macrophages, plays an important role in immune responses and systemic inflammation of the host. Moreover, interleukin-1*β*, produced by macrophages, participates in the immune response and tissue repair of the body. IL-1*β* can work in synergy with LL-37 to enhance the induction of specific inflammatory effectors by a complex mechanism involving multiple pathways, thus reinforcing selective immune responses of the host [37]. Baumgarner et al. [38] found that DIO mice exhibited a similar locomotor depression and selective impairments in their inflammatory response in both the periphery and brain following LPS administration. In the study, after infection, the levels of TNF-*α*, IL-6, and IL-1*β* in obese mice increased for a short time, and then decreased, which is conducive to reducing the inflammatory immune damage of the spleen and promoting the repair effect. The concentrations of these cytokines in normal mice increased and were maintained for a certain period of time, which will aggravate the inflammatory immune damage of the spleen.

The spleen is the site of initiation of most of the immune responses, and it can synthesize antibodies and filter foreign bodies and is involved in hematopoiesis [39]. Hence, the changes of the spleen structure affected the immune function of the host. Gulig et al. [9] found that the mice infected with *S. typhimurium* exhibited an increased number of large mononuclear cells in the white pulp, moderate necrosis of the red pulp including nuclear dust, and moderate inflammation and congestion of the red pulp. When the body was infected with *Salmonella*, the number of spleen cells significantly increased such as macrophages, lymphocytes, white blood cells, and red blood cells, which lead to the increase in spleen volume and weight [39]. However, studies on the spleen histopathology in nonfatal acute pneumonia caused by *E. coli* are limited. In this study, after infection, we found that MGC was recruited in the red pulp of the spleen in mice. The DIO-*E. coli* mice exhibited hyperemia in red pulp and lymphocytes were arranged loosely in white pulp at 12 h; the N-*E. coli* mice showed a serious hyperemia at 12 and 24 h; those helped to improve the spleen weight. The study suggested that spleen histopathological changes may be related to oxidative stress and inflammation levels after infection. Both parenchymal and capsular fibrosis can occur as a reparative process following injury or inflammation [40], which can maintain the relative integrity of tissue and organ structure. But sustained progress in fibrosis can lead to structural damage and dysfunction of organs [41]. The current study showed mild fibrous tissue of the spleen in DIO-*E. coli* and N-*E. coli* mice, and DIO-*E. coli* mice had more collagen fiber than N-*E. coli* mice. We considered that infection stimulated an immune response in the spleen and caused a certain degree of tissue damage; meanwhile, obesity exhibited a stronger tissue repair response and promoted the dissipation of inflammation, which may be related to oxidation and inflammation of the spleen.

## 5. Conclusions

Obesity elevated splenic inflammatory levels and decreased antioxidant capacity. Interestingly, the DIO mice exhibited a stronger antioxidant capacity than normal mice and the alleviation of inflammation in *E. coli* pneumonia. Compared with the normal mice, the DIO mice had increased resistance to spleen injury in the state of nonfatal infection. These results for the first time provided one possible view that obesity promoted immune responses of the spleen against pneumonia. However, the entire mechanisms are not yet clear and further study is needed.

## Data Availability

The data used to support the findings of this study are available from the corresponding author upon request.

## Abbreviations

DIO: Diet-induced obesity
*E. coli*: *Escherichia coli*
CFU: Colony-forming unit
GSH-Px: Glutathione peroxidase
SOD: Superoxide dismutase
CAT: Catalase
MDA: Malondialdehyde
TNF-*α*: Tumor necrosis factor-*α*
IL-6: Interleukin-6
IL-1*β*: Interleukin-1*β*.
